# CD8^+^ T cell activation occurs 24 hours after AAV administration and is driven by muscle promoter specificity

**DOI:** 10.1172/jci.insight.202541

**Published:** 2026-06-09

**Authors:** Lindsay Jeanpierre, Coralie Pecquet, Hanadi Saliba, Pauline Finard, Stéphane Terry, Gianni Tavella, Inès Guesmia, Sylvie Boutin, Bérangère Bertin, Sofia Benkhelifa-Ziyyat, Giuseppe Ronzitti, David-Alexandre Gross

**Affiliations:** 1 Généthon, Evry, France.; 2 Université Paris-Saclay, Université d’Évry, Inserm, Généthon, Integrare research unit UMR_S951, Evry, France.; 3 Research Department, Inovarion, Paris, France.; 4 Sorbonne Université, Inserm, Institut de Myologie, Centre de Recherche en Myologie, Paris, France.

**Keywords:** Immunology, Muscle biology, Gene therapy

## Abstract

Immune responses against transgene products can compromise adeno-associated virus–mediated (AAV-mediated) gene transfer. Although several factors influencing this immunogenicity have been described, the early in vivo events driving CD8^+^ T cell activation remain poorly defined. Here, we examined antigen presentation kinetics following intramuscular AAV administration in mice. Strikingly, viral genomes were detected in draining lymph nodes as early as 1 hour after injection, and transgene-derived peptides were presented to CD8^+^ T cells from day 1, resulting in progressive activation and first cell divisions detected at day 4. Removal of the injection site demonstrated that AAV particles reaching draining lymph nodes within the first hour were sufficient to induce cytotoxic transgene-specific CD8^+^ T cells. Finally, AAV vectors incorporating different muscle-specific promoters and regulatory sequences were evaluated. Although muscle specific, all promoters exhibited variable transgene expression in dendritic cells in vitro, correlating with early T cell activation in vivo; notably, those associated with higher early antigen presentation induced robust T cell response, whereas reduced presentation correlated with absence of CD8^+^ T cells. These findings reveal an unexpectedly early onset of transgene-derived epitope presentation, modulated by promoter specificity, which critically shapes CD8^+^ T cell response. This provides a rationale for evaluating and mitigating AAV immunogenicity in gene therapy design.

## Abstract 2

An adaptive immune response can develop as early as one day after AAV gene therapy and is influenced by how the therapeutic gene is regulated

## Graphical abstract

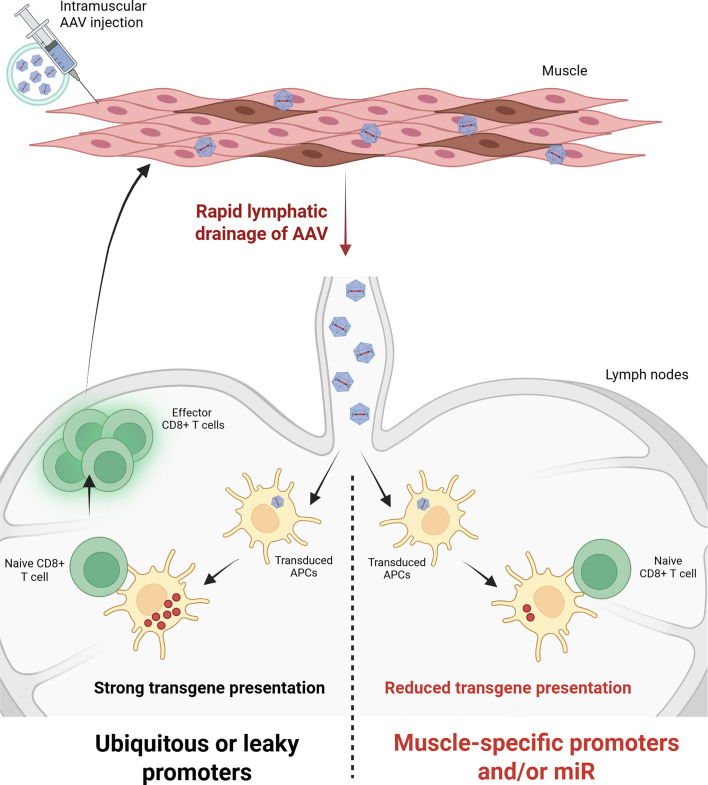

## Introduction

Recombinant adeno-associated virus (AAV) vectors are highly effective for expressing transgenes of therapeutic interest in specific target tissues. Over the years, strategies and vectors have been optimized to the extent that several AAV-based therapies have received regulatory approval for treating several genetic diseases, the most impressive one being onasemnogene abeparvovec, which has been used to treat thousands of spinal muscular atrophy patients. However, despite their relatively low inflammatory profile compared with other viral platforms such as adenoviral vectors, AAV vectors can still activate the immune system (1, 2). In addition to capsid-directed immunity, the immune response can target the transgene product itself, thereby compromising therapeutic efficacy through neutralization of the circulating transgene and/or rejection of the transduced cells (3, 4).

CD8^+^ T cell responses against the transgene product have long been documented in mouse models, a finding that initially prompted exploration of AAV vectors as potential vaccine platforms (5, 6), ultimately leading to their application during the COVID-19 pandemic (7, 8). However, such immune responses have rarely been reported in clinical trials. Notable examples include dystrophin-specific T cell responses reported in Duchenne muscular dystrophy (DMD) patients (9), follistatin-specific T cell responses in Becker muscular dystrophy (10), and anti–α_1_-antitrypsin (AAT) CD8^+^ T cells in AAT-deficient individuals (11). More recently, this issue has gained attention following reports of unexpected serious adverse events in 5 young DMD patients treated by 3 distinct AAV-microdystrophin products across separate clinical trials (12). According to preliminary laboratory findings, observed myositis and myocarditis were likely due to the cytotoxic T cell response (13). Indeed, the presence of a non-tolerized transgene-specific T cell repertoire appears to be a prerequisite for such reactions. In these AAV-microdystrophin trials, all 5 reactive patients harbored deletions encompassing exons 8–11, a region included in the microdystrophin transgene (12).

CD8^+^ T cells are primed within the secondary lymphoid organs by conventional dendritic cells (DCs), which are key professional antigen-presenting cells (APCs) (14). Depending on their maturation state and detection of pathogen-specific molecular patterns such as unmethylated CpG dinucleotides by Toll-like receptor 9, antigen presentation by DCs can result in either T cell tolerance or full activation and subsequent effector and memory T cell differentiation. Consistent with this, restricting transgene expression in APCs through AAV vectors engineered with muscle-restricted promoters has been shown to attenuate immune responses following AAV administration (15–17). Cordier and collaborators first demonstrated that replacing the ubiquitous CMV promoter with the muscle-specific MCK promoter enabled sustained expression of human γ-sarcoglycan in mice (15). The synthetic SPc5-12 muscle promoter showed mixed results, allowing sustained expression of human α-sarcoglycan in mice (16) and canine microdystrophin in dogs (17) but still causing anti–factor IX antibody formation in hemophilic mice (18). Hepatocyte-specific promoters have also been shown to promote immune tolerance in liver-directed gene transfer compared with ubiquitous promoters (19, 20). In addition to promoters, insertion of microRNA-142-3p (miR-142-3p) target sequences at the 3′ end of the transgene coding sequence effectively limits expression in APCs and promotes T cell tolerance following AAV-mediated muscle gene transfer in mice (21–25) and nonhuman primates (26). This strategy was recently refined by the combination of two microRNAs expressed in APCs but absent in muscle cells (27).

Classically, CD8^+^ T cell responses to AAV are assessed starting from day 7 after vector administration, when T cells are already activated and expanding. This timing implies that the initial antigen presentation events by APCs, which dictate the fate of the T cell response, occur several days earlier. Importantly, these early antigen presentation events are closely linked to promoter activity, which influences transgene expression levels in both transduced APCs and muscle cells. In this study, we investigated the location and kinetics of antigen presentation following intramuscular administration of AAV vectors in mice, focusing on the earliest time point at which transgene-specific CD8^+^ T cells can detect the antigen. We found that T cell activation occurs as early as 24 hours after injection and that muscle promoter specificity is a key determinant of this rapid response.

## Results

### Early and sustained transgene presentation to T cells after recombinant AAV administration.

**Figure 1:**
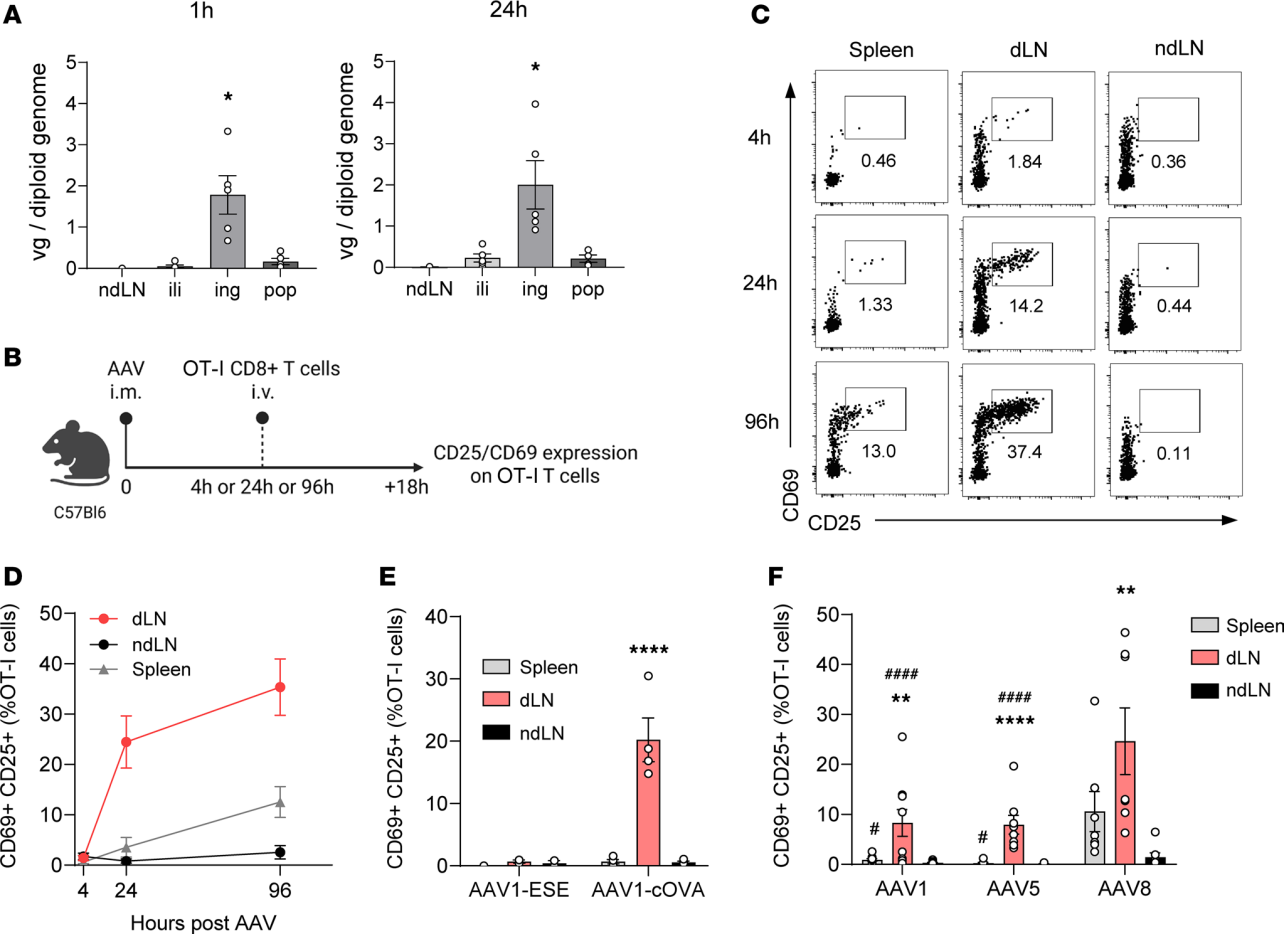
Early and sustained transgene presentation to T cells after recombinant AAV administration. (**A**) C57BL/6 mice (*n* = 5) were injected into the left tibialis anterior (TA) with 3 × 10^10^ vg of AAV1-CMV-luciferase. Left-side iliac (ili), inguinal (ing), and popliteal (pop) lymph nodes and right-side inguinal and popliteal lymph nodes (ndLN) were collected 1 or 24 hours after injection to perform AAV genome quantification by qPCR using transgene specific primers. (**B**) Experimental design for experiments in **C**–**F**: C57BL/6 mice were injected with 1 × 10¹^0^ vg of AAV into the left TA and subsequently received 1 × 10^6^ splenocytes from OT-I donor mice intravenously at the indicated time points. Spleen and left (draining; dLNs) and right (non-draining; ndLNs) pooled popliteal and inguinal lymph nodes were collected 18 hours after transfer and analyzed by flow cytometry. (**C** and **D**) As described in **B**, mice (*n* = 5) injected with AAV1-PGK-OVA_257_ received OT-I CD8^+^ T cells 4 hours, 24 hours, or 96 hours later. Representative dot plots (**C**) and kinetics (**D**) of the frequencies of CD69^+^CD25^+^ in live CD45.1^+^CD8^+^ OT-I T cells. (**E** and **F**) As described in **B**, mice (*n* = 4–10) were injected with either control AAV1-PGK-ESE or AAV1-PGK-cOVA (**E**) or different AAV serotypes (AAV1, AAV5, AAV8) encoding PGK-OVA_257_ (**F**) and received OT-I CD8^+^ T cells 24 hours later. Frequencies of CD69^+^CD25^+^ cells in live CD45.1^+^CD8^+^ OT-I T cells are represented. Each dot represents an individual mouse. Data are pooled from 2 (**A**), 3 (**B**–**D**), 1 (**E**), or 2 (**F**) independent experiments and are represented as mean ± SEM. Statistical significance was determined by 1-way ANOVA with Tukey’s multiple-comparison test: compared vs. ndLN (**A** and **F**) or dLN-AAV1-ESE (**E**), *P < 0.02 **P < 0.01, ****P < 0.0001; compared vs. AAV8, ^#^*P* < 0.02, ^####^*P* < 0.0001 (**F**).

To better characterize the kinetics and location of antigen presentation following AAV administration, C57BL/6 mice were injected intramuscularly into the left tibialis anterior with 3 × 10^11^ viral genomes (vg) of AAV1-CMV-luciferase and euthanized 1 hour or 24 hours later. Viral genomes were detected by qPCR as early as 1 hour after injection in lymph nodes draining the injection site (dLNs), with the highest levels observed in the left inguinal lymph node compared with the left popliteal and iliac lymph nodes (Figure 1A). As expected, no viral genomes were detected in the right contralateral non-draining popliteal and inguinal lymph nodes (ndLNs). Moreover, viral genome levels in dLNs remained unchanged at 24 hours, suggesting rapid lymphatic drainage rather than active transport by tissue-resident DCs (28, 29).

Unlike preformed protein antigens, transgenic proteins are produced only after AAV transduction, genome processing, and transgene expression. Consequently, presentation of transgene-derived peptides to CD8^+^ T cells is expected to occur later than that of exogenous proteins. To investigate this, we used the myotropic AAV1 vector, which exhibits limited liver transduction following intramuscular administration, thereby reducing the likelihood of liver-induced tolerance (30). The SIINFEKL peptide, placed under the control of the ubiquitous PGK promoter (AAV1-OVA_257_), was selected as the transgene, enabling CD8^+^ T cell activation to be analyzed independently of potential confounding factors such as anti-transgene CD4^+^ responses, antibody-mediated enhancement of CD8^+^ activation (23), or CD4^+^ regulatory T cells (31–33). After intramuscular administration of AAV1-OVA_257_, transgenic OVA_257_-specific CD8^+^ T cells (OT-I) were transferred intravenously at various time points. Their activation was assessed 18 hours later by the expression of the early activation markers CD25 and CD69 (Figure 1B). Strikingly, 24.5% ± 5.8% OT-I cells were activated in the dLNs as early as 24 hours after AAV administration, increasing to 35.3% ± 6.3% CD69^+^CD25^+^ at 96 hours (Figure 1, C and D). Activation was largely restricted to dLNs, with minimal activation detected in ndLNs and spleen.

We next asked whether this rapid presentation to T cells was specific to AAV1-OVA_257_ or represented a general feature of AAV vectors, regardless of encoded transgene or capsid serotype. First, we showed that the minimal OVA_257_ peptide transgene was not responsible for the rapid T cell activation. An AAV1 vector expressing cytoplasmic ovalbumin protein (cOVA), which is more representative of therapeutic intracellular transgenes and requires proteasomal processing before MHC class I presentation, also induced rapid OT-I activation in dLNs, whereas a control vector (AAV1-ESE) did not (Figure 1E). Furthermore, early CD8^+^ T cell activation was also observed in dLNs of mice injected with AAV5 and AAV8 vectors (Figure 1F). Notably, AAV8 induced greater activation in the dLNs and spleen compared with AAV1 and AAV5. As vector genome levels in the dLNs and spleen were comparable among serotypes (Supplemental Figure 1C; supplemental material available online with this article; https://doi.org/10.1172/jci.insight.202541DS1), these findings suggest serotype-dependent differences in APC transduction and/or transgene expression.

Finally, two additional control experiments were performed. First, we assessed the impact of injection volume: both lower (5 μL) and higher (100 μL) volume of AAV1-OVA_257_ resulted in similar OT-I activation in dLNs, suggesting that lymphatic drainage occurs independently of excessive muscle damage (Supplemental Figure 1A). Second, we verified that intact AAV particles were required for early presentation: before administration and OT-I transfer, AAV1-OVA_257_ vector and OVA_257_ peptide as control were heated to 95°C, a temperature that denatures AAV capsids and prevents transgene expression (34). As expected, heating the vector completely abolished OT-I activation, whereas heating the peptide had no effect (Supplemental Figure 1B).

Collectively, these findings demonstrate that AAV-encoded transgene is expressed and presented to T cells in the dLNs as early as 24 hours after administration.

### Early transgene presentation in dLNs drives efficient T cell activation.

**Figure 2:**
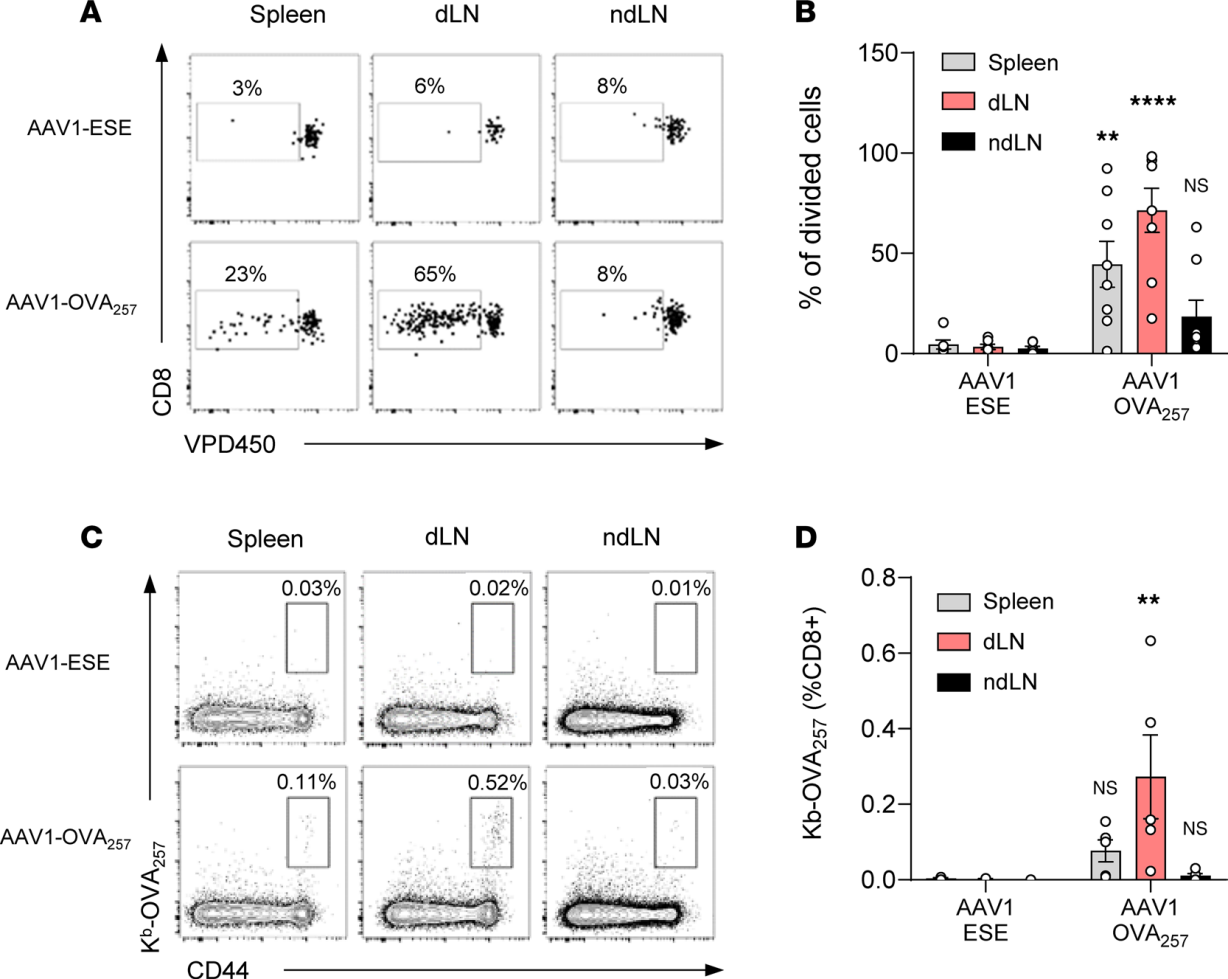
Early transgene presentation leads to efficient T cell activation. (**A** and **B**) C57BL/6 mice received intravenously 1 × 10^6^ VPD-labeled splenocytes from OT-I donor mice and 24 hours later were injected with 1 × 10^10^ vg of either AAV1-ESE (*n* = 6) or AAV1-OVA_257_ (*n* = 8) in the left TA (intramuscularly). On day 4, spleen, left dLNs (pooled popliteal and inguinal lymph nodes), and right ndLNs were collected and analyzed by flow cytometry. Representative dot plots (**A**) and frequencies (**B**) of VPD^lo^ cells in live CD45.1^+^CD8^+^ OT-I T cells. (**C** and **D**) C57BL/6 mice were injected in the left TA with 1 × 10^10^ vg of either AAV1-ESE (*n* = 3) or AAV1-OVA_257_ (*n* = 5). On day 7, spleen, left dLNs, and right ndLNs were collected and analyzed by flow cytometry. Representative dot plots (**C**) and frequencies (**D**) of K^b^OVA_257_ tetramer^+^ CD44^+^ cells in live CD8^+^ cells. Each dot represents an individual mouse. Data are pooled from 3 (**A** and **B**) or 2 (**C** and **D**) independent experiments and are represented as mean ± SEM. Statistical significance was determined by 1-way ANOVA with Tukey’s multiple-comparison test: comparison vs. AAV1-ESE, **P < 0.01, ****P < 0.0001.

Upregulation of early activation markers such as CD25 and CD69 reflects antigen encounter and does not necessarily indicate complete T cell activation and efficient clonal expansion. To assess this and follow initial T cell divisions, we tracked Violet Proliferation Dye–labeled (VPD-labeled) OT-I cells transferred 1 day before AAV1-OVA_257_ administration. As early as day 4, 71.4% ± 11.7% of OT-I cells had divided and accumulated in the dLNs and 44.5% ± 12.2% in the spleen, whereas few divided cells were present in the ndLNs and none in mice receiving the AAV1-ESE control vector (Figure 2, A and B). Importantly, in the absence of non-physiological OT-I cell transfer, we also detected at day 7 endogenous OVA_257_-specific CD8^+^ T cells that were effectively activated by AAV1-OVA_257_ and expanded (Figure 2, C and D). Under these conditions, 0.3% ± 0.1% of tetramer^+^ CD8^+^ T cells were found in the dLNs and 0.1% ± 0.05% in the spleen, versus 0% in ndLNs or after AAV1-ESE administration.

**Figure 3:**
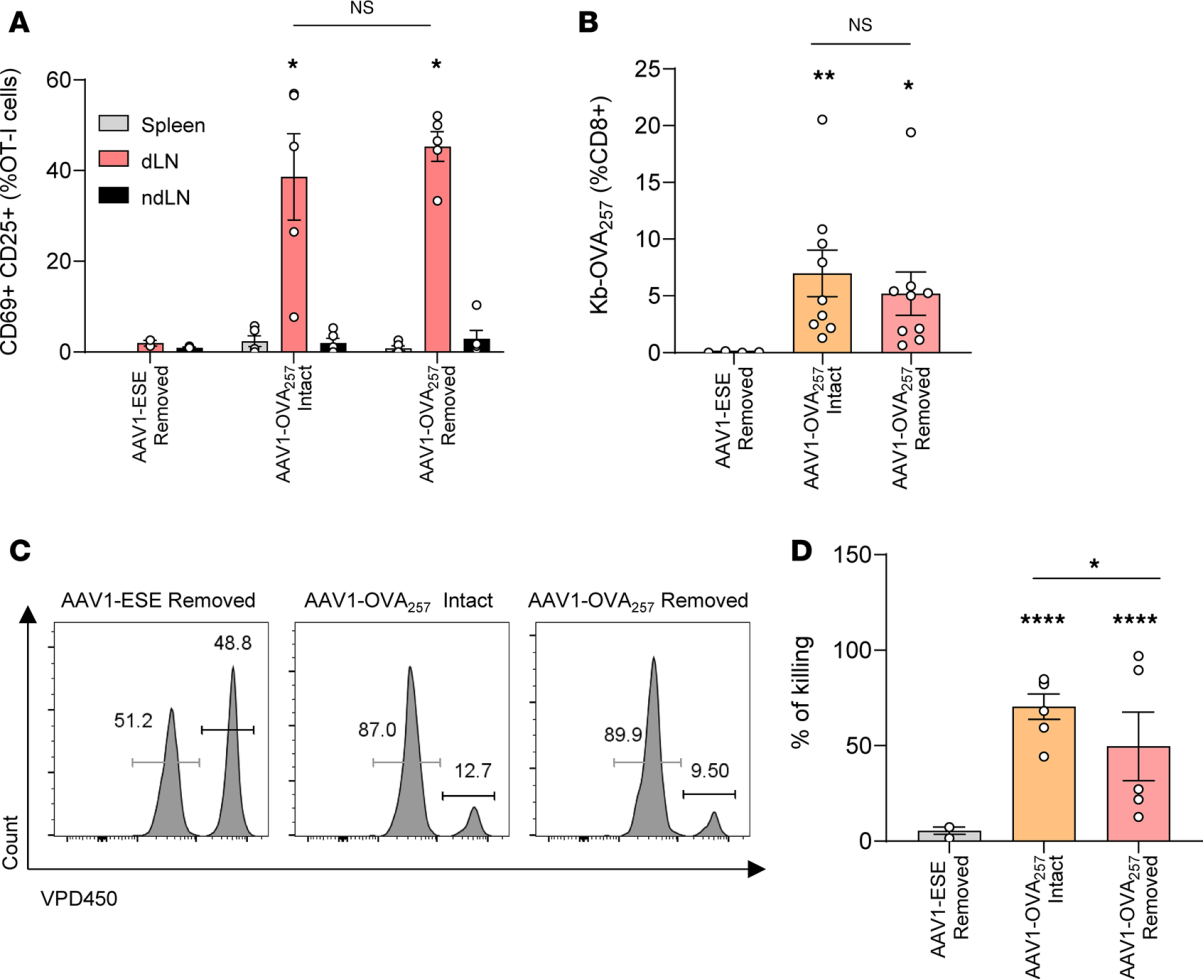
Early transgene presentation is sufficient to induce a specific CD8^+^ response. (**A**–**D**) C57BL/6 mice were injected intradermally in the top of the ear with 1 × 10^10^ vg of either AAV1-ESE or AAV1-OVA_257_. One hour later, the top of the ear was excised (Removed) or left intact (Intact). Different experiments were performed after this treatment and are represented in the corresponding panels. (**A**) Twenty-four hours after excision, mice (*n* = 3–5) received intravenously 1 × 10^6^ VPD-labeled splenocytes from OT-I donor mice and were euthanized 18 hours later. Spleen, superficial cervical lymph nodes (parotid and mandibular; dLNs), and popliteal and inguinal lymph nodes (ndLNs) were collected and analyzed by flow cytometry. Histogram showing the frequencies of CD69^+^CD25^+^ in live CD45.1^+^CD8^+^ OT-I T cells. (**B**) At 2 weeks after AAV, mice (*n* = 4–9) were bled to analyze K^b^OVA_257_ tetramer^+^ CD44^+^ cells in live CD8^+^ cells by flow cytometry. (**C** and **D**) Six weeks after ear excision, mice received through intravenous injection a mixture of unloaded and OVA_257_-loaded splenocytes (1 × 10^7^ total cells). Mice (*n* = 3–6) were euthanized 2 days later, and spleens were collected and analyzed by flow cytometry. (**C**) Representative histograms gated on live B220^+^ donor splenocytes. (**D**) Percentage of cytotoxicity. Each dot represents an individual mouse. Data are pooled from 3 (**A**–**D**) independent experiments and are represented as mean ± SEM. (**A**) Statistical significance was determined by 1-way ANOVA with Tukey’s multiple-comparison test: comparison vs. AAV1-ESE, *P < 0.1. (**B**–**D**) Statistical significance was determined by the Kruskal-Wallis test with Dunn’s multiple-comparison test, *P < 0.1, **P < 0.01, ****P < 0.0001.

Next, we investigated whether AAV particles reaching the dLNs via lymphatic drainage — rather than active transport by DCs — were sufficient for efficient T cell activation. To this end, AAV1-OVA_257_ was injected intradermally into the top of the ear, a route previously shown to be at least as immunogenic as the intramuscular one (35). One hour later, the injection site was excised to ensure that only rapidly drained AAV particles remained (29). As expected, OT-I cells were activated in dLNs to a similar extent after ear excision versus in intact mice, confirming rapid lymphatic drainage of AAV particles (Figure 3A). No nonspecific activation was observed after ear excision upon administration of AAV1-ESE vector (Figure 3A). Critically, in a separate series of experiments without OT-I T cell transfer, we found that this early antigen encounter was sufficient to drive robust endogenous CD8^+^ T cell response (Figure 3B). As shown by tetramer staining, OVA_257_-specific CD8^+^ T cells were efficiently expanded despite removal of the injection site, with 5.2% ± 2.0% tetramer^+^ among CD8^+^ T cells, versus 7.0% ± 2.2% in intact mice, at day 14 after vector injection (Figure 3B). To test whether these CD8^+^ T cells induced after injection site removal were functional, an in vivo cytotoxic assay was performed by transfer of OVA_257_ peptide–loaded splenocytes in mice previously injected with AAV. Six weeks after AAV administration, VPD450^hi^ OVA_257_ peptide–loaded splenocytes were equally rejected by mice with intact or excised injection sites (Figure 3, C and D).

Altogether, these results demonstrate that the AAV particles reaching the dLNs within the first hour after injection are sufficient to optimally prime and expand functional cytotoxic CD8^+^ T cells.

### Muscle-specific expression reduces early antigen presentation and T cell activation.

**Figure 4:**
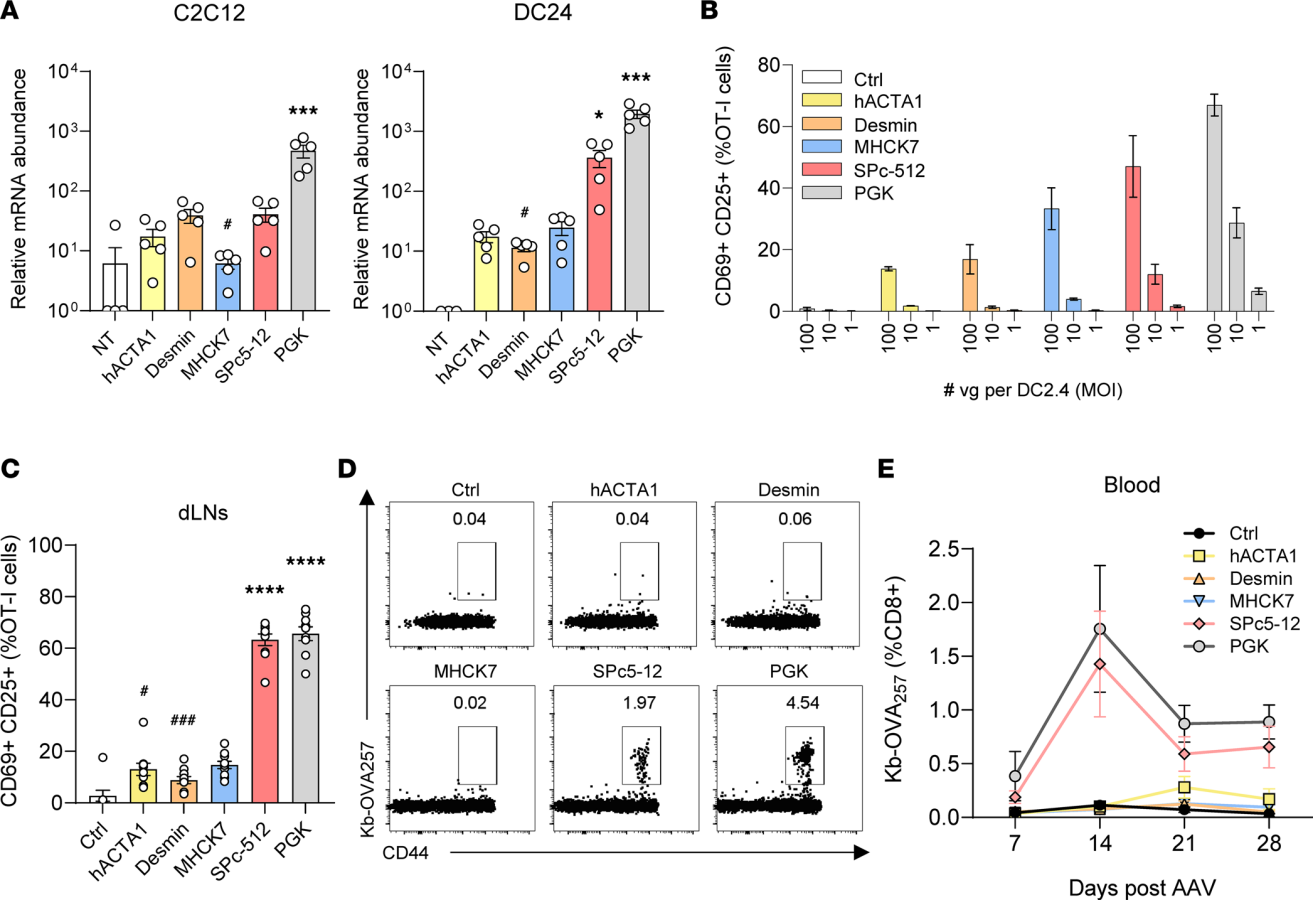
Muscle-specific promoters reduce early antigen presentation and CD8^+^ T cell responses. (**A**) Murine myoblast C2C12 cells or dendritic DC2.4 cells were transduced by 1 × 10^5^ vg/cell of different AAV1 vectors encoding OVA_257_ under the control of indicated promoters (NT, not transduced). Transgene mRNA was quantified by specific RT-qPCR 24 hours after transduction. (**B**) DC2.4 cells were transduced with the indicated MOIs of the different AAV1-OVA_257_ vectors for 24 hours, and cocultured with CD8^+^ OT-I T cells for 18 hours. Expression of CD69/CD25 activation markers on CD8^+^ OT-I T cells was assessed by flow cytometry. (**C**) C57BL/6 mice (*n* = 8–10) were injected with 1 × 10^10^ vg of indicated AAV1-OVA_257_ vectors in the left TA 24 hours before receiving 1 × 10^6^ splenocytes from OT-I donor mice through intravenous injection. Left popliteal and inguinal dLNs were collected 18 hours later and analyzed by flow cytometry. Frequencies of CD69^+^CD25^+^ in live CD45.1^+^CD8^+^ OT-I T cells are represented. (**D** and **E**) C57BL/6 mice (*n* = 8) were injected in the left TA with indicated AAV1-OVA_257_ vectors and were bled at indicated time points to measure transgene-specific CD8^+^ T cells by flow cytometry. Representative dot plots (**D**) and kinetics (**E**) showing the frequencies of KbOVA_257_ tetramer^+^ CD44^+^ cells in live CD8^+^ cells. Each dot represents an independent experiment (**A** and **B**) or an individual mouse (**C**–**E**). Data are pooled from 2 (**A** and **B**) or 3 (**C** and **E**) independent experiments and are represented as mean ± SEM. Statistical significance was determined by the Kruskal-Wallis test with Dunn’s multiple-comparison test: comparison vs. Ctrl, *P < 0.1, ***P < 0.001, ****P < 0.0001; comparison vs. SPc5-12, ^#^*P* < 0.01, ^###^*P* < 0.001.

Given the importance of early antigen presentation for T cell priming, we wondered whether its reduction would be beneficial for limiting unwanted T cell responses. To this end, we sought to restrict transgene expression to muscle cells, thereby limiting expression in APCs and reducing early transgene-derived antigen presentation. We generated five AAV1 vectors encoding the OVA_257_ epitope, each driven by a different muscle-specific promoter. Their expression was confirmed in murine C2C12 myoblasts, by RT-qPCR analysis 24 hours after transduction (Figure 4A). Unexpectedly, transgene expression was also detected in DC2.4 murine dendritic cells (DCs) for all promoters. Expression levels were comparable for SPc5-12 and the ubiquitous PGK promoter, with SPc5-12 tending to induce a higher expression than the other muscle-specific promoters (Figure 4A). To determine whether this promoter leakiness in cultured DCs resulted in effective transgene presentation to CD8^+^ T cells, DC2.4 cells were transduced with the different AAV1 vectors at different MOI for 24 hours, then cocultured with OT-I T cells. Promoters enabled OT-I T cell activation with SPc5-12 and PGK promoters appearing stronger than human ACTA1 (hACTA1), desmin, or MHCK7, in agreement with RT-qPCR results (Figure 4B). These in vitro findings were confirmed in vivo using the previously described approach: intramuscular AAV administration followed by assessment of OT-I activation at 24 hours. Except desmin, for which the effect was not significant, all tested muscle-specific promoters supported antigen presentation in the dLNs, with SPc5-12 and PGK showing higher levels compared with hACTA1, desmin, and MHCK7 (Figure 4C). Comparable results were obtained in the spleen (Supplemental Figure 2A). This differential antigen presentation in lymphoid organs is not explained by differences in transduction efficiency or transgene expression, as equivalent levels of vector genome copy number and transgene mRNA were measured in muscle (Supplemental Figure 2, B and C). Overall, mRNA expression levels in DC2.4 cells were strongly correlated with antigen presentation both in vitro and in vivo (Supplemental Figure 2, D and E). Importantly, promoter leakiness resulting in elevated antigen presentation both in vitro and in vivo correlated with robust priming of transgene-specific CD8^+^ T cells. Thus, endogenous OVA_257_-specific CD8^+^ T cells were primed and expanded only following administration of SPc5-12 and PGK AAV1 vectors (Figure 4, D and E).

**Figure 5:**
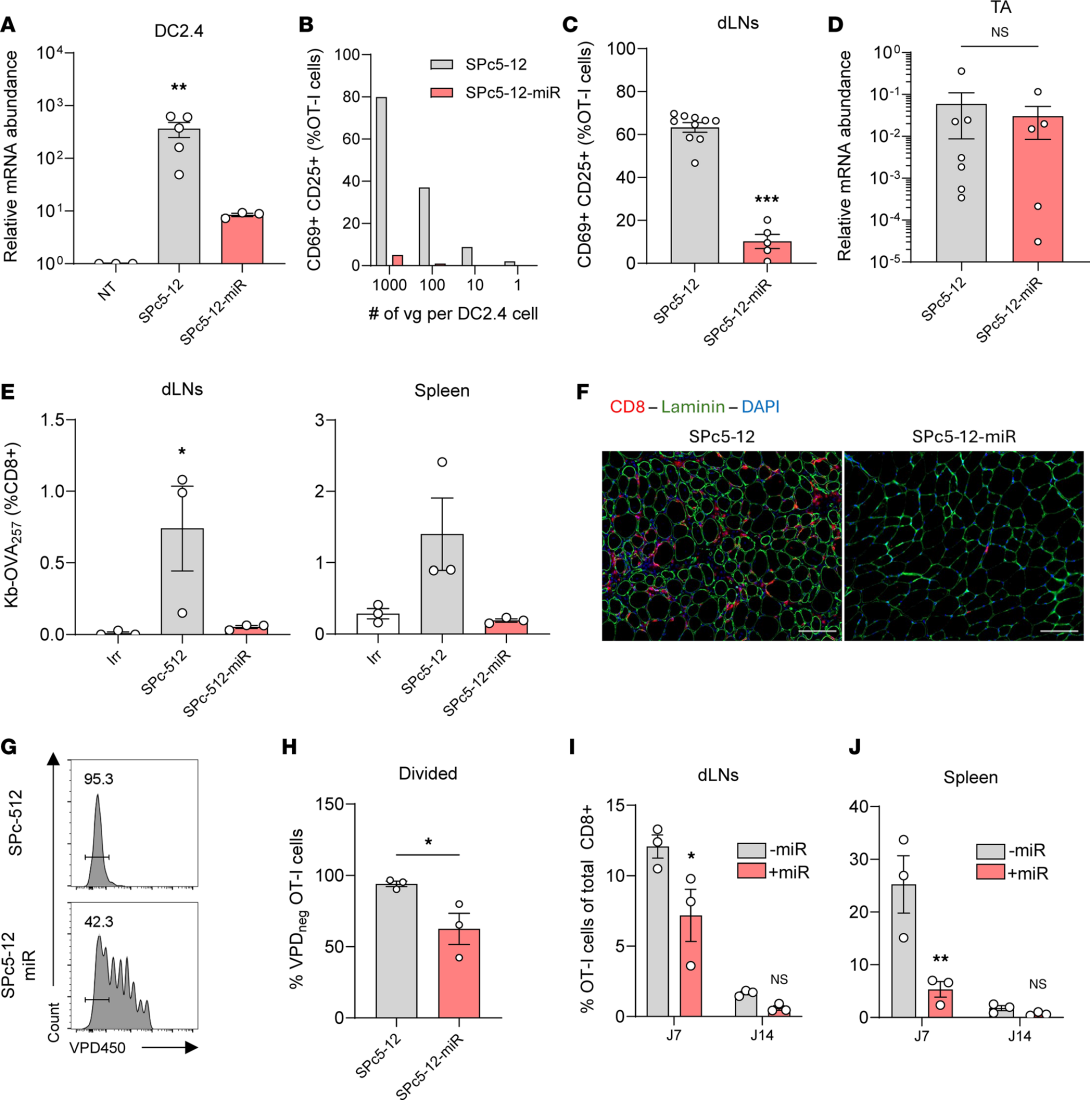
Enforced muscle-specific expression inhibits proper CD8^+^ T cell activation. (**A**) Transgene mRNA was quantified by specific RT-qPCR 24 hours after transduction of DC2.4 cells by 1 × 10^5^ vg/cell of indicated AAV1-OVA_257_ vectors. (**B**) DC2.4 cells were transduced with indicated MOIs of the different AAV1-OVA_257_ vectors for 24 hours, and cocultured with CD8^+^ OT-I T cells for 18 hours. Expression of CD69/CD25 on CD8^+^ OT-I T cells was assessed by flow cytometry. (**C**–**J**) C57BL/6 mice were injected intramuscularly in the left TA with 1 × 10^10^ vg of either AAV1–SPc5-12–OVA_257_, AAV1–SPc5-12–OVA_257_–miR, or an irrelevant vector (Irr) encoding murine SEAP under the control of the PGK promoter. (**C** and **D**) Twenty-four hours after AAV, mice (*n* = 5–10) received intravenously 1 × 10^6^ splenocytes from OT-I donor mice. dLNs and TA were collected 18 hours later. (**C**) Histogram showing the frequencies of CD69^+^CD25^+^ in live CD45.1^+^CD8^+^ OT-I T cells. (**D**) Transgene mRNA levels in the left TA were quantified by RT-qPCR. (**E** and **F**) Two weeks after AAV, dLNs and spleen were collected and analyzed by flow cytometry (*n* = 3). (**E**) Frequencies of K^b^OVA_257_ tetramer^+^ CD44^+^ cells in live CD8^+^ cells. (**F**) Immunofluorescence staining of left TA for CD8 (red), laminin (green), and DAPI (blue). Scale bars: 100 μM. (**G**–**J**) Mice (*n* = 3) received 1 × 10^5^ VPD-labeled splenocytes from OT-I donor mice before AAV injection in the left TA (1 × 10^10^ vg). Proliferation of OT-I cells in dLNs at day 7 (**G** and **H**) and frequency in spleen and dLNs at indicated time points (**I** and **J**) were assessed by flow cytometry. Each dot represents an individual mouse. Data are pooled from 3 (**A**, **C**, and **D**) or 1 (**B**, **E**–**J**) independent experiments and are represented as mean ± SEM. Statistical analysis: (**A** and **E**) Kruskal-Wallis test with Dunn’s multiple-comparison test: vs. Ctrl or Irr, *P < 0.1, **P < 0.01. (**C**, **D**, and **H**) Mann-Whitney *U* test: *P < 0.1, ***P > 0,0001. (**I** and **J**) 2-way ANOVA: vs. SPc5-12; *P < 0.1, **P < 0.01.

Leakiness of SPc5-12 promoter was previously reported in plasmacytoid DCs (36). To limit its nonspecific activity in APCs, we inserted miR-142-3p target sequences into the SPc5-12 cassette, a strategy known to restrict transgene mRNA expression in hematopoietic cells (37). In vitro, this miR-based regulation reduced transgene mRNA expression in the DC2.4 cell line, as well as the activation of OT-I T cells (Figure 5, A and B). Consistently, in vivo transgene presentation at 24 hours was diminished, as evidenced by lower OT-I activation in mice receiving AAV1–SPc5-12–miR vector compared with those receiving AAV1–SPc5-12 vector (Figure 5C). This reduction could not be explained by decreased transgene expression in muscle, since equivalent levels were measured by RT-qPCR (Figure 5D). Importantly, in separate experiments, OVA_257_-specific CD8^+^ T cells were not detected in lymphoid organs (dLNs and spleen) 14 days after AAV1–SPc5-12–miR vector administration (Figure 5E), nor in blood during a 1-month follow-up (data not shown). Moreover, CD8^+^ T cell infiltration in the tibialis anterior was considerably reduced in mice injected with AAV1–SPc5-12–miR vector as compared with those injected with AAV1–SPc5-12 (Figure 5F). Consistently, muscle fiber integrity was preserved by the addition of the miR-142-3p target sequences. To further assess the fate of OVA_257_-specific CD8^+^ T cells, we transferred 1 × 10^5^ VPD-labeled OT-I cells prior to AAV vectors and followed their proliferation over time. In mice injected with miR-regulated AAV vectors, OT-I cells showed reduced proliferation (Figure 5, G and H), resulting from limited expansion in both dLNs and spleen at day 7 and no accumulation thereafter (Figure 5, I and J).

Altogether, our results indicate that enforcing muscle-specific expression reduces early transgene presentation and prevents efficient CD8^+^ T cell activation.

## Discussion

CD8^+^ T cell response directed against the transgene product represents a major barrier for long-term efficacy of AAV-based gene therapy, and previous works have identified numerous parameters shaping this response. In this study, we focused on the earliest steps of the CD8^+^ T cell activation by characterizing the location and kinetics of antigen presentation following intramuscular AAV administration. Our findings revealed unexpectedly rapid transgene presentation in the draining lymph nodes and a correlation between these early events and efficient CD8^+^ T cell priming.

Viral genomes were observed in the lymph nodes draining the injected tibialis anterior (TA) as early as 1 hour after AAV administration. Interestingly, viral genome counts were higher in inguinal than in popliteal lymph nodes, suggesting that the latter are not the primary lymph node draining the TA. Moreover, transgene presentation was not affected by removal of the injection site 1 hour after injection. Given their small size (~25 nm), AAV particles can reach the lymph node in two ways: actively by APCs migrating from the TA, or passively through the lymphatic system. Active transport by tissue-resident DCs typically requires 16 hours to several days, depending on the DC subset (28, 29), whereas passive lymphatic drainage of small particles, such as mRNA vaccines or virions, occurs within minutes, leading to antigen deposition into the lymph node subcapsular sinus (38–40). This allows the relatively few naive antigen-specific T cells to be activated without delay during an infection. Our findings strongly support passive lymphatic drainage of AAV particles. However, we were unable to visualize AAV capsids by immunofluorescence or immunohistochemistry in draining lymph node sections, and whether AAV particles interact with APCs in the subcapsular sinus or penetrate deeper into the T cell zone remains to be determined.

Transgene-derived peptides were presented to CD8^+^ T cells as early as 24 hours after injection. While rapid capsid capture and presentation by APCs were anticipated, the unexpectedly early onset of transgene expression and presentation was striking. This phenomenon was observed for both a minimal OVA_257_ epitope and the full-length cytoplasmic OVA protein, as well as for an MHC class II epitope (data not shown), excluding the implication of a possible transgene construct artifact. Previous studies reported detectable factor IX expression in human myoblasts and myotubes only 2–3 days after transduction (41), and in the blood 3 days after AAV1 administration (42), with 30%–90% of maximal expression achieved at this time point with AAV8 or AAV9 vectors (43). This might indicate that the transgene is expressed for many hours but still not detected by conventional assays. Indeed, detection of the transgenic protein depends not only on its level of expression but also on the sensibility of the detection methods (Western blot, ELISA, or fluorescence). Given that T cells can be activated by very few peptide-MHC complexes (44), they represent an exquisitely sensitive tool for detection of minimal transgene expression.

The benefit of tissue-restricted promoters and miR-based regulation in reducing immune responses has been demonstrated for muscle targeting (15–17, 21–24) and liver-directed gene transfer (19, 20, 37, 45). This has led to the use of muscle-specific promoters, namely MHCK7, SPc5-12, MSP, and CK8, in current AAV-microdystrophin clinical trials (12). Consistent with fundamental immunological principles, avoiding direct transgene expression in APCs is an obvious strategy (14). Interestingly, the fact that mRNA-LNP vaccines — another gene therapy with radically opposite goals — are in part based on transduction of DCs is also an illustration of this rationale. Nevertheless, AAV transduction of APCs, particularly DCs, and the subsequent transgene expression remain under scrutiny. Initial reports suggested poor AAV transduction of murine and human DCs in vitro (46, 47), but a later study challenged these findings (48). In vivo evidence indicates that DC transduction does occur (46, 49, 50), although at limited levels (51, 52). This limited transduction is underscored by ongoing efforts to engineer AAV vectors with enhanced dendritic cell tropism for immunization purposes (53–55). In our study, detection of DC transduction within hours of injection was technically challenging, likely owing to the low proportion of transduced cells and minimal early transgene expression. Nonetheless, our data using muscle-specific promoters and miR-142-3p regulation support the importance of limiting early presentation, i.e., hematopoietic cell transduction. Notably, the SPc5-12 promoter, previously shown to be expressed in human APCs (36), failed to reduce the initial antigen presentation in our study and elicited a robust CD8^+^ T cell response.

Using a single MHC class I epitope allowed us to assess CD8^+^ responses independently of CD4^+^ help, antibodies, or regulatory T cells. Our results demonstrate that this minimal transgene is sufficient to prime and expand fully cytotoxic CD8^+^ T cells. Previous studies reported dysfunctional CD8^+^ response following intramuscular AAV administration, characterized by expansion and cytotoxic potential, but poor capacity to be restimulated by the antigen and to eliminate transduced cells (56–58). Whether CD8^+^ T cells elicited by AAV1-OVA_257_ differ mechanistically was not addressed here. Several parameters may play a role. Large volumes of injection (50–100 μL) can extend beyond muscle tissue, potentially leading to liver transduction and tolerance induction, particularly with non-muscle-restricted promoters (58). Additionally, regulatory T cells may exert a detrimental effect on CD8^+^ T cells when transgenes contain potential MHC class II epitopes, as shown in the context of AAV gene transfer in both mice (31, 32) and humans (33). Moreover, the recognition of AAV genomes by TLR9, leading to CD8^+^ T cell activation through type I interferon (59, 60), provides a rationale for the observed benefit of CpG motif reduction in humans (61). In our study, the vector contained very few CpG motifs owing to the small size of the transgene, making it unlikely that early antigen presentation was mediated by TLR9 signaling. However, APC maturation and the capacity of APCs to prime effector T cells are surely impacted by innate sensing, and it would require further investigations when using muscle-specific expression to reduce immune response.

Finally, this study focuses on local AAV delivery, as it remains the best-established and prototypical system for investigating immune responses to the transgene product. However, systemic administration has now become the preferred approach in current clinical trials aiming to target the largest possible number of muscles, using more appropriate AAV serotypes such as AAV9 or AAVrh74 (62). Using systemic delivery with these clinically relevant vectors, we similarly detected early presentation of the transgene to CD8^+^ T cells, indicating that this phenomenon is not restricted to local delivery but represents a more general principle (data not shown). The extent to which leakiness and tissue-restricted expression (i.e., muscle-specific expression) modulate the CD8^+^ T cell response under these conditions is currently under investigation.

Thus, beyond conferring tissue-specific transgene expression, muscle-restricted promoters and miR-based regulation effectively attenuate immune responses against the transgene product. Our findings uncover an unexpectedly early phase of transgene presentation to T cells that critically influences the efficiency of T cell priming. Incorporating assays that quantify these initial antigen presentation events in APCs into current immunogenicity assessment would provide valuable insight into the design of AAV vectors. Moreover, such approaches represent an important step forward in elucidating CD8^+^ T cell responses to systemic AAV delivery in both liver- and muscle-targeted gene therapy.

## Methods

### Sex as a biological variable.

Our study exclusively examined female mice. Because sex-dependent variations in transduction efficacy or transgene expression have been described in various tissues, such as the liver (63), brain (64, 65), lungs (66), and eye (67), we confirmed in male mice our main results, i.e., the early presentation in dLNs, as well as the leakage of the various muscle promoters and its prevention by miR-142-3pT sequences (Supplemental Figure 3).

### Mice.

Six- to eight-week-old female C57BL/6N mice were purchased from Charles River Laboratories (Le Genest Saint Isle, France). Rag2^–/–^ OT-I (CD45.1) mice carrying a TCR specific for OVA_257_ bound to H-2K^b^ and C57BL/6N CD45.1 congenic mice were bred in our animal facility. Mice were housed under specific pathogen–free conditions at the Centre d’exploration et de recherche fonctionnelle expérimentale (CERFE, Evry, France) and handled in accordance with French and European directives. For intramuscular or intradermal injections, mice were anesthetized with a mix of ketamine (100 mg/kg) and xylazine (10 mg/kg), and 25 μL of indicated AAV vector diluted in phosphate-buffered saline (PBS) was injected into the tibialis anterior or the ear dermis using a 30-gauge RN Hamilton syringe. For blood sampling and intravenous injections, mice were anesthetized with isoflurane (4%).

### Plasmid construction and recombinant AAV vector production.

All plasmids were codon optimized and generated using the pSMD2 backbone, with the SV40 polyadenylation signal. Nucleotide sequences of promoters, transgenes, and regulatory elements used in this study are provided in Supplemental Table 1. A small DNA tag was inserted at the 3′ end of the coding sequence to allow DNA and mRNA quantification. The SIINFEKL peptide (OVA_257_) sequence was placed under the control of either the ubiquitous PGK promoter or muscle-specific human ACTA1, desmin, MHCK7, or SPc5-12 promoters. The AAV1-ESE control vector encodes a scrambled sequence under the control of a U7 promoter (68). The irrelevant control vector (Irr) is an AAV1 encoding the murine SEAP under the control of the ubiquitous PGK promoter. Recombinant AAV vectors were produced by triple transfection, and vector particles were purified by affinity column as described before (69). Titers were determined by qPCR amplification, using specific primers for the polyadenylation signal or the DNA tag.

### Quantification of viral genomes and transgene mRNA.

For viral genome quantification, left (injected) and right (non-injected) tibialis anterior muscles were collected and directly frozen into MN Bead tubes (Machery-Nagel). After addition of 500 μL of PBS, tissues were homogenized by mechanical shaking with 4 movements per second for 45 seconds, using the Fisherbrand Bead Mill 24 homogenizer. A fraction of the cell suspension was used to extract DNA with a NucleoMag Pathogen kit (Machery-Nagel) using a KingFisher instrument (KingFisher Flex, Thermo Fisher Scientific). qPCR was performed on the DNA extracts to quantify the viral genomes in the muscles, with polyA-SV40 and housekeeping titin-specific primers, and estimate the viral genome copy number per cell (diploid genome). For mRNA extraction on tissue, a fraction of the homogenized muscle was preserved in Nucleozol (Macherey-Nagel). mRNA was extracted with NucleoMag RNA Pro (Macherey-Nagel) using a Labgene-32 instrument (Labgene Scientific). cDNA was generated with the RevertAid H Minus First Strand cDNA Synthesis Kit (Thermo Fisher Scientific).

Transgene mRNA quantification was performed after in vitro transduction of murine cell lines. Mouse C2C12 myoblasts and dendritic cell line DC2.4 were cultivated in sterile conditions, at 37°C, 5% CO_2_, respectively in DMEM and RPMI medium, supplemented with 10% FBS and 1% penicillin-streptomycin. To prevent C2C12 myoblast differentiation, cells were maintained under high-serum growth conditions (10% FBS) and kept below 60%–70% confluence by passaging every 2 days. Cells were transduced in vitro with SIINFEKL-expressing AAV1 vectors, at different MOI ranging from 1 × 10^4^ to 1 × 10^6^ vg/cell, and were collected 24 to 48 hours later. Total RNA was purified using a miRNeasy Micro Kit (QIAGEN), and cDNA was generated with the RevertAid H Minus First Strand cDNA Synthesis Kit. Transgene mRNA relative expression was evaluated after qPCR using primers specific for the DNA tag and the P0 housekeeping gene.

### Cell isolation and transfer.

Lymphoid organ cell suspensions were prepared from immunized recipients by mechanical dissociation of spleen, pooled (or individual) left popliteal and inguinal lymph nodes (dLNs) or right ones (ndLNs), or superficial cervical (parotid and mandibular; ear-draining) lymph nodes in sterile PBS containing 0.1% HSA. For T cell proliferation assay, OT-I T cell suspensions were stained in PBS containing 2 μM Violet Proliferation Dye 450 (VPD450) (BD Biosciences) for 8 minutes at 37°C, followed by 2 washes in PBS containing first 2% HSA and then 0.1% HSA. For in vivo cytotoxic assay, naive splenocytes from C57BL/6N CD45.1 mice loaded or not with 10 μM OVA_257_ epitopes were incubated respectively with 0.2 μM or 2 μM VPD450 (BD Biosciences) at 37°C for 10 minutes. After washing, the cells were mixed at a 50:50 ratio, and 1 × 10^7^ cells were injected intravenously. Cell suspensions were injected into the retro-orbital venous sinus in a final volume of 200 μL of PBS.

### Flow cytometry analysis.

For peripheral blood lymphocyte staining, erythrocytes were first eliminated by hypotonic shock with BD Pharm Lyse buffer (BD Biosciences). Stainings were performed in PBS containing 0.1% BSA. Cell suspensions were incubated with anti-FcγRIII/II (2.4G2, Bio X Cell) mAb for 15 minutes at 4°C and then stained for 30 minutes at 4°C with saturating amounts of given combinations of the following antibodies: FITC– or e450–anti-CD8α (eBioscience, 53-6.7), FITC–anti-CD44 (BioLegend, IM7), APC–anti-TCRβ (BD Pharmingen, H57-597), PE– or PE-Cy7–anti-CD69 (BioLegend, H1-2F3), APC–anti-CD25 (BioLegend, PC61), PE-Cy7–anti-CD45.1 (BioLegend, A20), AF700–anti-CD45.2 (BioLegend, 104), PE–anti-CD45R (eBioscience, B220). For tetramer staining, an initial PE–H-2K^b^/OVA_257_ tetramer (CliniSciences) staining was performed in PBS containing 0.1% HSA for 30 minutes at room temperature. For in vivo cytotoxic assay, splenocytes were labeled with PE–anti-CD45R to analyze for VPD450 expression in B cells. The percentage of specific lysis of cognate (c) over nonspecific/cognate cells (u) was calculated for each mouse (i) in comparison with naive mice (n) as follows: % specific lysis = [(c/u)n − (c/u)i]/[(c/u)n] × 100. Dead cells were excluded using 7-actinomycin D (Sigma-Aldrich) or LIVE/DEAD Fixable Near-IR staining kit (Life Technologies). Cells were finally fixed with 2% paraformaldehyde for 15 minutes and kept at 4°C, and data were collected using a Beckman Coulter CytoFLEX S flow cytometer and further analyzed using FlowJo software v10.9 (Waters Corporation).

### Statistics.

All data are shown as mean ± standard error of the mean (SEM). All statistical analyses were performed using GraphPad Prism Software version 10. Specific tests used for each analysis are indicated in the respective figure legends.

### Study approval.

All procedures were approved by the local ethics committee (CEEA-051, Evry, France) and authorized by the French Ministry of Research (MESRI) under numbers 2019-019 #24963 and 2022-016 #39963.

### Data availability.

All individual values represented in graphs are provided in the Supporting Data Values XLS file or otherwise are available upon request.

## Author contributions

LJ, CP, HS, PF, ST, GT, IG, SB, and BB performed experiments. LJ, CP, and HS analyzed and prepared figures. SBZ provided AAV5 and AAV8 vectors. LJ, GR, and DAG wrote the manuscript.

## Conflict of interest

The authors have declared that no conflict of interest exists.

## Funding support

- Association Française contre les Myopathies (AFM).
- Agence Nationale de la Recherche (ANR-24-CE17-7239-01).
- Paris Ile-de-France Region (DIM Thérapie Génique).
- Action Thématique Incitative de Genopole (ATIGE).
- European Union (Horizon Europe project 101080690–MAGIC).
- French Ministry of Research (to LJ, PF, and IG).
- Programme et Équipement Prioritaire de Recherche (PEPR) QUALAAV, funded by the French government under the France 2030 investment plan and managed by the ANR (to GT).

## Supplementary Material

Supplemental data

Supporting data values

## Version 1 06/09/2026

In-Press Preview

## Version 2 07/22/2026

Electronic publication
