# Supplementary material for: CD8+ T cell activation occurs 24 hours after AAV administration and is driven by muscle promoter specificity
Source: JCI Insight. 2026 Jun 9;11(14):e202541. doi: 10.1172/jci.insight.202541 (PMC13461172; doi:10.1172/jci.insight.202541)
Supplement: Supplementary file 1 [file jciinsight-11-202541-s018.pdf]

## Supplementary Table 1

| | |
|---|---|
| **$OVA_{257}$** | AGTATAATCAACTTTGAAAAACTG |
| **hACTA1** | AAAGGCATAGCCCCATATATCAGTGATATAAATAGAACCTGCAGCAGGCTCTGGTAAATGATGACTACAAGGTGGACTGGGAGGCAGCCCGGCCTTGGCA<br>GGCATCGACCGGGCCAACCCGCTCCTTCTTTGGTCAACGCAGGGGACCCGGGCGGGGGCCCAGGCCGCGAACCGGCCGAGGGAGGGGGCTCTAGTGCCC<br>AACACCCAAATATGGCTCGAGAAGGGCAGCGACATTCCTGCGGGGTGGCGCGGAGGGAATGCCCGCGGGCTATATAAAACCTGAGCAGAGGGACAAGC<br>GGCCACCGCAGCGGACAGCGCCAAGTG |
| **Desmin** | TACCCCCTGCCCCCCACAGCTCCTCTCCTGTGCCTTGTTTCCCAGCCATGCGTTCTCCTCTATAAATACCCGCTCTGGTATTTGGGGTTGGCAGCTGTTGCTGC<br>CAGGGAGATGGTTGGGTTGACATGCGGCTCCTGACAAAACACAAACCCCTGGTGTGTGTGGGCGTGGGTGGTGTGAGTAGGGGGATGAATCAGGGAGG<br>GGGCGGGGGACCCAGGGGGCAGGAGCCACACAAAGTCTGTGCGGGGGTGGGAGCGCACATAGCAATTGGAAACTGAAAGCTTATCAGACCCTTTCTGGA<br>AATCAGCCCACTGTTTATAAACTTGAGGCCCCACCCTCGACAGTACCGGGGAGGAAGAGGGCCTGCACTAGTCCAGAGGGAAACTGAGGCTCAGGGCTAG<br>CTCGCCCATAGACATACATGGCAGGCAGGCTTTGGCCAGGATCCCTCCGCCTGCCAGGCGTCTCCCTGCCCTCCCTTCCTGCCTAGAGACCCCCACCCTCAA<br>GCCTGGCTGGTCTTTGCCTGAGACCCAAACCTCTTCGACTTCAAGAGAATATTTAGGAACAAGGTGGTTTAGGGCCTTTCCTGGGAACAGGCCTTGACCCTT<br>TAAGAAATGACCCAAAGTCTCTCCTTGACCAAAAAGGGGACCCTCAAACTAAAGGGAAGCCTCTCTTCTGCTGTCTCCCCTGACCCCACTCCCCCCCACCCCA<br>GGACGAGGAGATAACCAGGGCTGAAAGAGGCCCGCCTGGGGGCTGCAGACATGCTTGCTGCCTGCCCTGGCGAAGGATTGGCAGGCTTGCCCGTCACAG<br>GACCCCCGCTGGCTGACTCAGGGGCGCAGGCCTCTTGCGGGGGAGCTGGCCTCCCCGCCCCCACGGCCACGGGCCGCCCTTTCCTGGCAGGACAGCGGGA<br>TCTTGCAGCTGTCAGGGGAGGGGAGGCGGGGGCTGATGTCAGGAGGGATACAAATAGTGCCGACGGCTGGGGGCCCTGTCTCCCCTCGCCGCATCCACT<br>CTCCGGCCGGCCGCCTGCCCGCCGCCTCCTCCGTGCGCCCGCCAGCCTCGCCCGC |
| **MHCK7** | AAGCTTGCATGTCTAAGCTAGACCCTTCAGATTAAAAATAACTGAGGTAAGGGCCTGGGTAGGGGAGGTGGTGTGAGACGCTCCTGTCTCTCCTCTATCTG<br>CCCATCGGCCCTTTGGGGAGGAGGAATGTGCCCAAGGACTAAAAAAAGGCCATGGAGCCAGAGGGGCGAGGGCAACAGACCTTTCATGGGCAAACCTTG<br>GGGCCCTGCTGTCTAGCATGCCCCACTACGGGTCTAGGCTGCCCATGTAAGGAGGCAAGGCCTGGGGACACCCGAGATGCCTGGTTATAATTAACCCAGA<br>CATGTGGCTGCCCCCCCCCCCCCAACACCTGCTGCCTCTAAAAATAACCCTGTCCCTGGTGGATCCCCTGCATGCGAAGATCTTCGAACAAGGCTGTGGGGG<br>ACTGAGGGCAGGCTGTAACAGGCTTGGGGGCCAGGGCTTATACGTGCCTGGGACTCCCAAAGTATTACTGTTCCATGTTCCCGGCGAAGGGCCAGCTGTC<br>CCCCGCCAGCTAGACTCAGCACTTAGTTTAGGAACCAGTGAGCAAGTCAGCCCTTGGGGCAGCCCATACAAGGCCATGGGGCTGGGCAAGCTGCACGCCT<br>GGGTCCGGGGTGGGCACGGTGCCCGGGCAACGAGCTGAAAGCTCATCTGCTCTCAGGGGCCCCTCCCTGGGGACAGCCCCTCCTGGCTAGTCACACCCTG<br>TAGGCTCCTCTATATAACCCAGGGGCACAGGGGCTGCCCTCATTCTACCACCACCTCCACAGCACAGACAGACACTCAGGAGCAGCCAGC |
| **PGK** | CCACGGGGTTGGGGTTGCGCCTTTTCCAAGGCAGCCCTGGGTTTGCGCAGGGACGCGGCTGCTCTGGGCGTGGTTCCGGGAAACGCAGCGGCGCCGACCC<br>TGGGTCTCGCACATTCTTCACGTCCGTTCGCAGCGTCACCCGGATCTTCGCCGCTACCCTTGTGGGCCCCCCGGCGACGCTTCCTGCTCCGCCCCTAAGTCGG<br>GAAGGTTCCTTGCGGTTCGCGGCGTGCCGGACGTGACAAACGGAAGCCGCACGTCTCACTAGTACCCTCGCAGACGGACAGCGCCAGGGAGCAATGGCA<br>GCGCGCCGACCGCGATGGGCTGTGGCCAATAGCGGCTGCTCAGCGGGGCGCGCCGAGAGCAGCGGCCGGGAAGGGGCGGTGCGGGAGGCGGGGTGTG<br>GGGCGGTAGTGTGGGCCCTGTTCCTGCCCGCGCGGTGTTCCGCATTCTGCAAGCCTCCGGAGCGCACGTCGGCAGTCGGCTCCCTCGTTGACCGAATCACC<br>GACCTCTCTCCCCAG |
| **SPc5-12** | CCACCGCGGTGGCGGCCGTCCGCCCTCGGCACCATCCTCACGACACCCAAATATGGCGACGGGTGAGGAATGGTGGGGAGTTATTTTTAGAGCGGTGAGG<br>AAGGTGGGCAGGCAGCAGGTGTTGGCGCTCTAAAAATAACTCCCGGGAGTTATTTTTAGAGCGGAGGAATGGTGGACACCCAAATATGGCGACGGTTCCT<br>CACCCGTCGCCATATTTGGGTGTCCGCCCTCGGCCGGGGCCGCATTCCTGGGGGCCGGGCGGTGCTCCCGCCCGCCTCGATAAAAGGCTCCGGGGCCGGC<br>GGCGGCCCACGAGCTACCCGGAGGAGCGGGAGGCGCCAAGCTCTAGAACTAGTGGATCT |
| **miR142-3pT** | TCCATAAAGTAGGAAACACTACACGATATTCCATAAAGTAGGAAACACTACAACCGGTTCCATAAAGTAGGAAACACTACATCACACTCCATAAAGTAGGA<br>AACACTACA |
| **DNA Tag** | TGACCTCACGCAATCTTCTGACTGCGGACCTGCAGGAGGAGGCAGCGGTGGTGGAGAATACCCACGCACCACC |

**Supplementary Table 1**

Nucleotide sequences of promoters, transgenes, and regulatory elements used in this study.

**Supplemental Figure 1**

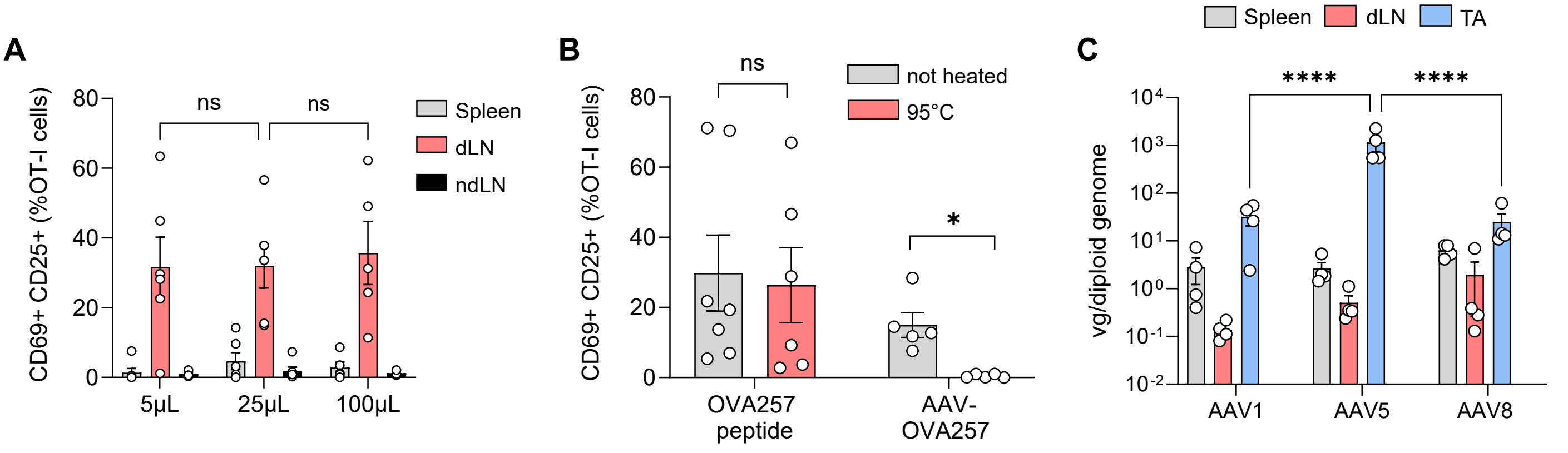


**Supplemental Figure 1**

(**A**) C57BL/6 mice (n=5-6) were administered i.m. in the left TA with in 5, 25 or 100µl containing 1x$10^{10}$ vg of AAV1-$OVA_{257}$, then received intravenously 1x$10^{6}$ splenocytes from OT-I donor mice 24h later. Spleen, left pooled popliteal and inguinal lymph nodes (dLNs) and right pooled popliteal and inguinal lymph nodes (ndLNs) were collected 18h later and analyzed by flow cytometry. Histogram shows the frequencies of CD69+ CD25+ in live CD45.1+ CD8+ OT-I T cells.
(**B**) Mice were treated as described in A but were administered with either intact or heated AAV1-$OVA_{257}$ vector (1x$10^{10}$vg), intact or heated $OVA_{257}$ peptide (50ng). Frequencies of CD69+ CD25+ in live CD45.1+ CD8+ OT-I T cells are represented.
(**C**) Mice (n=4) were treated as described in Figure 1F and spleen, left pooled popliteal and inguinal lymph nodes (dLNs), right pooled popliteal and inguinal lymph nodes (ndLNs) and TA muscles were collected 24h post injection to perform AAV genome quantification by qPCR using transgene-specific primers.
Each dot represents an individual mouse. Data are pooled from 2 (A) or 3 (B) independent experiments and are represented as mean ± SEM. Statistical significance was determined by Two-way Anova : (ns) non-significant, *p<0.1.

**Supplemental Figure 2**

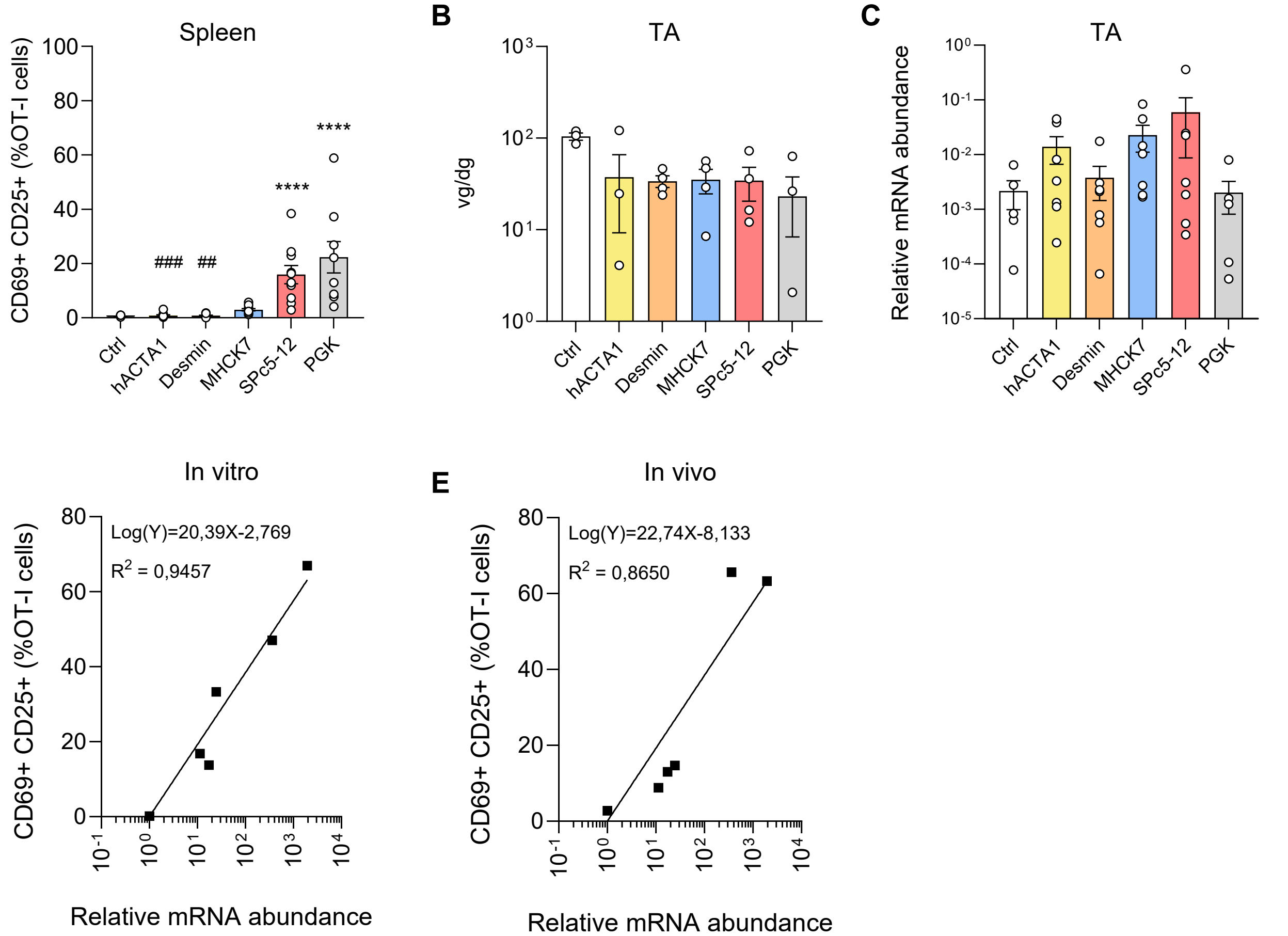


**Supplemental Figure 2**

(**A-C**) Mice (n=8-10) were treated as described in Figure 4C. (**A**) Frequencies of CD69+ CD25+ in live CD45.1+ CD8+ OT-I T cells in the spleen. (**B**) Viral genome copy number per diploid genome (vg/dg) in left TA determined by qPCR using transgene-specific primers. (**C**) Relative transgene mRNA abundance in left TA quantified by RT-qPCR 24h post transduction. (**D-E**) Linear regression was performed between relative mRNA abundance in DC2.4 cells (data from Fig.4A) and frequencies of CD69+ CD25+ OT-I cells in vitro (**D** – data from Fig.4B) or in vivo (**E** – data from Fig.4C). Each dot represents one of the previously described promoters.

Each dot represents an individual mouse. Data are pooled from 3 independent experiments and are represented as mean ± SEM. Statistical significance was determined by the Kruskal–Wallis test with Dunn's multiple-comparisons test : comparison versus Ctrl ****p<0.0001 ; comparison versus spC512, $^{###}$p<0.00, $^{####}$p<0.0001.

**Supplemental Figure 3**

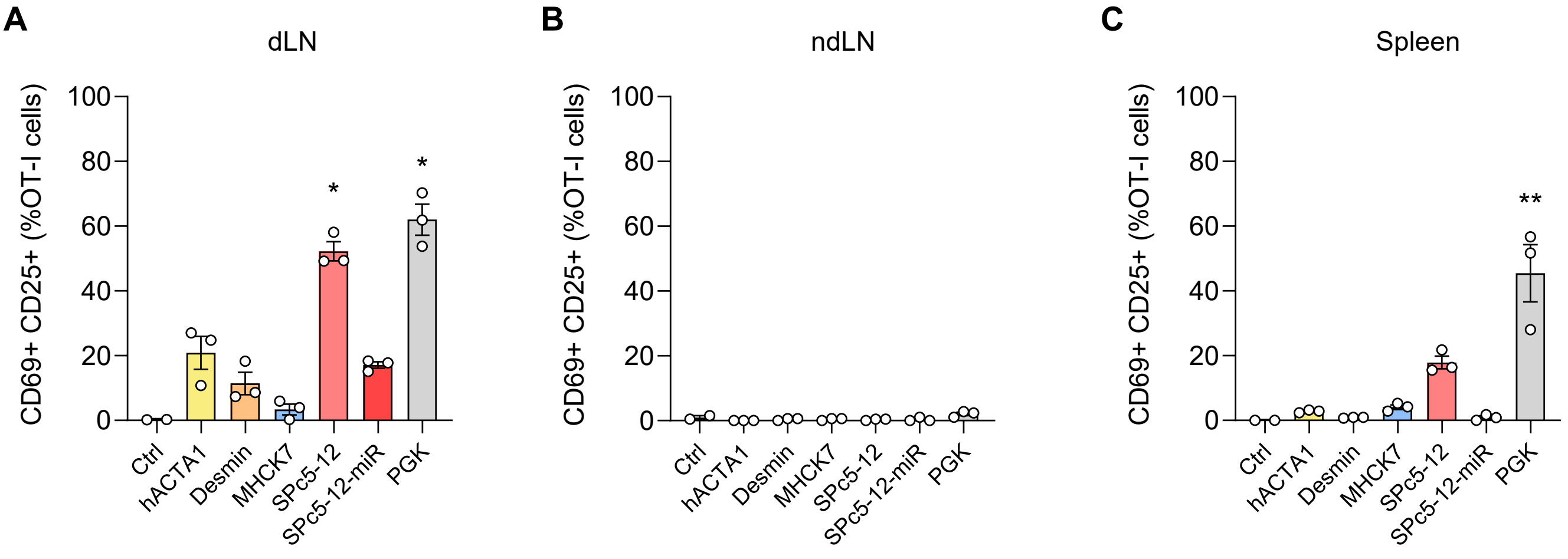


**Supplemental Figure 3**

(**A-C**) C57BL/6 male mice (n=3) received 1x10$^{10}$vg of indicated vectors in the left TA 24h prior transfer with 1x10$^{6}$ splenocytes from OT-I donor mice. **(A)** Left (dLNs) and **(B)** right (ndLNs) pooled popliteal and inguinal draining lymph nodes and (**C**) spleen were collected 18h later and analyzed by flow cytometry. Histograms show the frequencies of CD69+ CD25+ in live CD45.1+ CD8+ OT-I T cells.

Each dot represents an individual mouse. Data are represented as mean ± SEM. Statistical significance was determined by the Kruskal–Wallis test with Dunn's multiple-comparisons test : comparison versus Ctrl, *p<0.1, **p<0.01.
